# 3D- and 4D-Printed Nano-Enabled Soft Robots for Cancer Diagnosis and Therapy: Materials, Manufacturing, Image Guidance, and Translational Validation

**DOI:** 10.3390/bios16100546

**Published:** 2026-09-29

**Authors:** Ye Ri Han, Sang Bong Lee

**Affiliations:** 1 Department of Chemistry, Duksung Women’s University, Seoul 01369, Republic of Korea; 2 SimVista Inc., A-13, 194-25 Osongsaengmyeong 1-ro, Osong-eup, Heungdeok-gu, Cheongju 28160, Republic of Korea; 3 Department of Biomedical Sciences, Chonnam National University Medical School, 264 Seoyang-ro, Hwasun 58128, Republic of Korea; 4 Department of Life Science, Dongguk University-Seoul, 32 Dongguk-ro, Ilsandong-gu, Goyang 10326, Republic of Korea

**Keywords:** 3D and 4D printing, additive manufacturing, biomedical robotics, cancer theranostics, nanocomposite materials, soft robotics

## Abstract

Three-dimensional (3D) and four-dimensional (4D) printing expand the design space for nano-enabled soft robots intended for cancer diagnosis and therapy. This structured narrative review separates three evidence classes: core systems satisfying all scope criteria, adjacent technologies satisfying only part of the definition, and enabling technologies not yet integrated as oncology soft robots. The revised evidence set contains 12 core reports, 7 adjacent comparators and 68 enabling methods, standards or background reports. Study-level analysis using independent manufacturing, biological, workflow and safety (M/B/W/S) axes shows that the core literature is dominated by single-platform, in vitro therapeutic demonstrations with absent or short-term safety evidence (S0-S1). No core study established cancer-specific diagnostic sensitivity or specificity, no human validation was identified, and only two systems were classified as genuine or candidate 4D platforms under a strict programmed-transformation definition. Hydrogel, magnetic, photothermal, conductive, imaging-active and melanin-based materials are evaluated together with printability, dose, localization, sterilization, retrieval, degradation and nanoparticle fate. Translation requires reproducible manufacture, quantitative application endpoints, exposure-matched safety testing and comparison with established oncology workflows. The principal gap is not the availability of additional functions, but the lack of integrated evidence connecting controlled manufacture to clinically meaningful diagnosis or therapy. These findings apply only to the studies identified within the predefined search and eligibility framework and do not establish the absence of relevant evidence elsewhere.

## 1. Introduction

Cancer diagnosis and therapy repeatedly confront the same engineering problem: a spatially confined and mechanically heterogeneous target must be reached, characterized, and treated while limiting injury to surrounding tissue. Foundational soft robotics studies established distributed compliance and unconventional actuation [1,2], whereas additive manufacturing reviews showed how print paths, multimaterial interfaces, channels, and anisotropy can encode function [3,4,5]. More recent analyses extend these concepts to biomedical and nano-enabled systems but also expose unresolved trade-offs among print fidelity, force, payload, image visibility, and biological safety [6,7]. Accordingly, the present review evaluates what printing and nanoscale phases contribute to an oncology workflow rather than treating every soft or small robot as equivalent evidence. To support cross-platform comparison, the review links material formulation and printing architecture to independent manufacturing, biological, workflow, and safety evidence axes.

In this review, a nano-enabled soft robot is an additively manufactured, compliant and controllable device in which an intentionally incorporated nanoscale phase is necessary for at least one core function—actuation, sensing, imaging, heating, transport, adhesion, or drug release. The definition does not require the whole robot to be nanometer-sized, and it excludes a printed soft structure bearing an incidental nanomaterial that is not functionally validated. Because most candidate devices are micro- to millimeter-scale, size, payload, force, visibility, recovery, and clearance must be reported separately from the nanoscale mechanism. Generic nanoparticles, unprinted nanocarriers, passive phantoms, and rigid surgical robots are outside the core evidence set and are cited only as explicitly labeled comparators.

Three-dimensional printing denotes layerwise additive fabrication of a stable architecture. In this review, 4D printing is reserved for a printed structure whose geometry, permeability, mechanical state, spatial exposure, or function changes after printing through a programmed material–architecture response to time and a defined stimulus. Time-dependent drug diffusion alone, externally imposed movement of a passive printed body, or the mere presence of a responsive polymer is insufficient; the printed design must encode the transformation, and the response must be measured for magnitude, kinetics, repeatability, and reversibility where relevant [8,9,10].

Research on this topic is distributed across soft robotics, additive manufacturing, nanomedicine, imaging, and interventional oncology. This review therefore distinguishes (i) core systems, in which additive manufacturing defines the active robot body, programmed nanocomposite distribution, therapeutic microstructure, or patient-specific interface; (ii) adjacent additively manufactured comparators, in which printing supplies tooling or passive/nonconductive components; and (iii) nonprinted benchmarks, included only to compare propulsion, imaging, release, or heating. This boundary prevents platform proximity from being mistaken for evidence that a nano-enabled printed robot has performed a clinically defined task.

### 1.1. Literature Search and Review Methodology

This article was prepared as a structured narrative review rather than a systematic review or meta-analysis. PubMed, Web of Science Core Collection, Scopus and Google Scholar were searched for publications from 1 January 2000 through 31 August 2026. Database-specific Boolean strategies are provided in Table 1. Search concepts covered additive manufacturing (3D print*, 4D print*, direct ink writing, stereolithography, digital light processing and two-photon polymerization), soft or miniature robotics, nanoparticles or nanocomposites, and oncology-relevant functions (cancer, tumor, biopsy, diagnosis, imaging, drug delivery, hyperthermia, chemoembolization and theranostics). Backward and forward citation searches were used to identify foundational and recent studies.

Eligible studies had to report an additively manufactured compliant robotic body with a functionally necessary nanoscale phase and an experimentally demonstrated oncology-relevant function to enter the core evidence set. Reports satisfying only part of this definition were retained as adjacent comparators, while nonrobotic materials, printing methods, standards and nanomedicine studies were classified as enabling evidence. The revised reference set comprises 87 publications: 12 core reports, 7 adjacent printed or clinical comparator reports and 68 enabling methods, standards or background reports. Titles and abstracts were screened by one author, followed by full-text assessment against the stated criteria. This single-reviewer process may have introduced selection bias. Heterogeneous designs and endpoints precluded quantitative pooling; evidence was therefore synthesized using material–process–function relationships, study-level M/B/W/S assessment and translational endpoints.

Database searches, explicit eligibility criteria and study-level classification were used to structure the narrative synthesis; these features do not make it an exhaustive systematic review. The search interval, selected databases, terminology and restrictive eligibility criteria may have excluded relevant studies, and single-reviewer screening introduces a risk of selection bias. Consequently, throughout this review, statements that no study or no qualifying level of evidence was identified mean that no such study was identified within the predefined search and eligibility framework, rather than that no such evidence exists in the wider literature.

**Figure 1:**
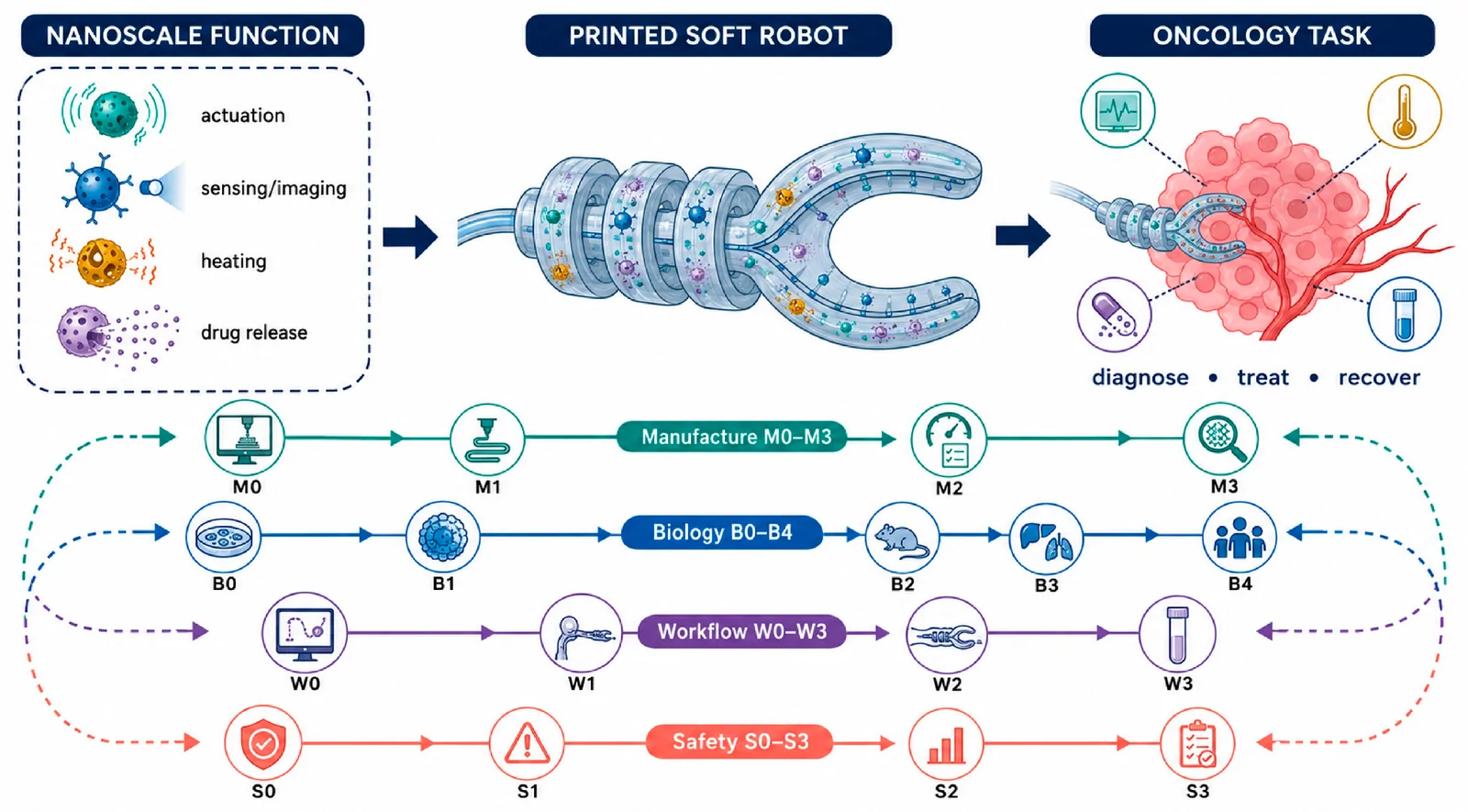
Multiscale scope and oncology-specific evaluation loop. A functional nanoscale phase is incorporated into a printed compliant body and linked to a defined diagnostic or therapeutic task. Manufacturing, biological, workflow and safety evidence should be reported independently. Solid navy arrows show the progression from nanoscale function to the printed robot and oncology task. The lower color-coded paths denote independent manufacturing (teal), biological (blue), workflow (purple), and safety (red) evidence axes; dashed return arrows indicate reassessment of those axes. Colors distinguish functions and evidence domains, not measured performance. Conceptual schematic; no experimental, patient or primary research data are shown. AI tool and version: OpenAI ChatGPT (version 5.6). Prompt used: “Create a clean, publication-ready biomedical infographic on a white background showing a left-to-right pathway from nanoscale functions (actuation, sensing/imaging, heating, and drug release), to a 3D-printed compliant soft robot, to an oncology task involving diagnosis, treatment, and recovery. Beneath the pathway, show four independent, color-coded evidence axes: manufacturing M0–M3, biology B0–B4, workflow W0–W3, and safety S0–S3. Use precise scientific icons, navy headings, restrained teal, blue, purple, and orange accents, and no experimental or patient data.” The authors reviewed and edited the generated output, verified the scientific content, and take full responsibility for this figure.

**Figure 2:**
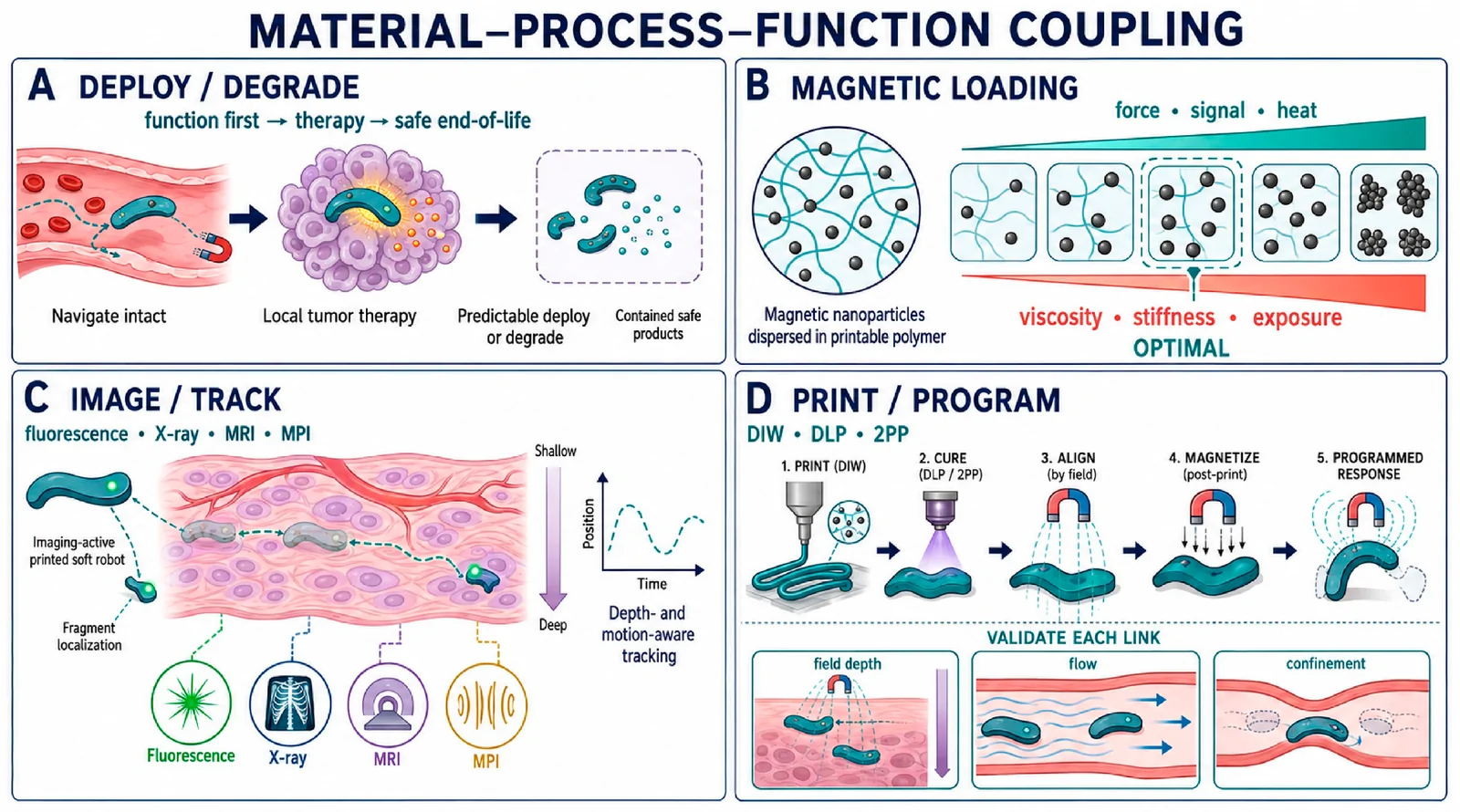
Material–process–function map for nano-enabled printed soft robots. Material choice, printing route and programmed response form a coupled design problem; each connection requires study-specific validation. Arrows indicate conceptual process sequences in panels A and D, tracking depth and time in panel C, and flow in the validation example. In panel B, increasing nanoparticle loading is accompanied by competing functional benefits and material or exposure constraints; the wedges are qualitative, not quantitative scales. Conceptual schematic; no experimental, patient or primary research data are shown. AI tool and version: OpenAI ChatGPT (version 5.6). Prompt used: “Create a four-panel scientific schematic titled Material-Process-Function Coupling for nano-enabled printed soft robots. (**A**) should show intact navigation, local tumor therapy, predictable deployment or degradation, and safe containment. (**B**) should show increasing magnetic nanoparticle loading and the trade-offs among force, imaging signal, heating, viscosity, stiffness, and exposure. (**C**) should compare fluorescence, X-ray, MRI, and MPI for intact-robot and fragment tracking with depth and time. (**D**) should show printing as programming through print, cure, align, magnetize, and programmed response, validated under field depth, flow, and confinement. Use a clean white-background biomedical infographic style.” The authors reviewed and edited the generated output, verified the scientific content, and take full responsibility for this figure.

**Figure 3:**
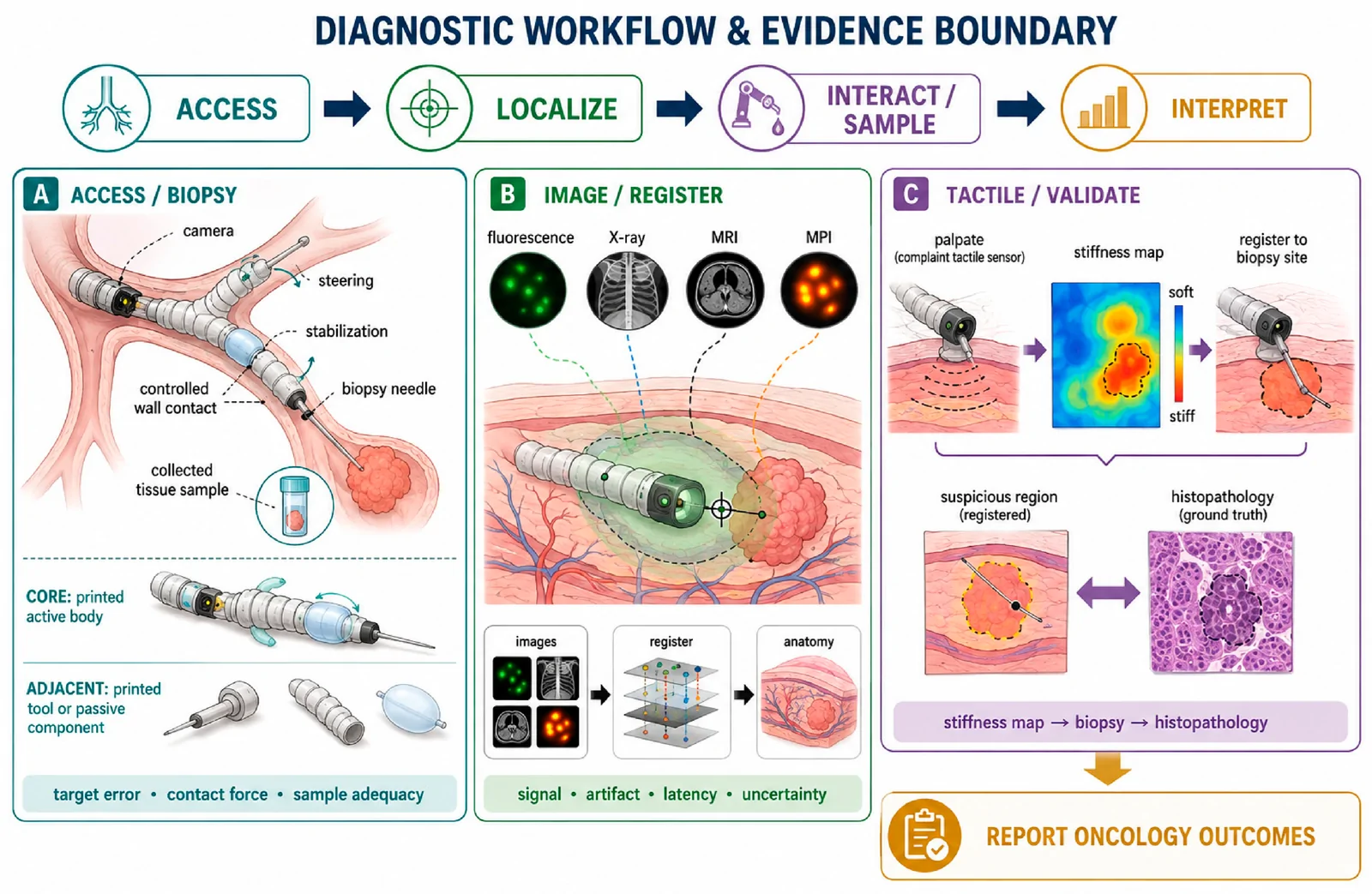
Diagnostic workflow and evidence boundary. Core printed systems and adjacent additively manufactured comparators can inform lesion access, localization, sampling and mechanical sensing, but application claims require oncology-specific outcome measures. Solid arrows indicate the diagnostic sequence and panel-specific registration or validation steps. Dashed colored lines connect imaging modalities to the same anatomical target, and dotted outlines indicate the illustrated region of interest or tissue section. Colored bright spots depict illustrative modality-dependent signals, not measured lesions or nanoparticle counts. The blue-to-red stiffness map denotes a qualitative soft-to-stiff scale; histopathology provides the proposed reference for validation. Conceptual schematic; no experimental, patient or primary research data are shown. AI tool and version: OpenAI ChatGPT (version 5.6). Prompt used: “Create a publication-ready three-panel biomedical infographic titled Diagnostic Workflow and Evidence Boundary. Across the top, show the sequence access, localize, interact/sample, and interpret. (**A**) should depict a steerable compliant soft robotic biopsy device in an airway, controlled wall contact, a biopsy needle, and collected tissue, while distinguishing a core printed active body from adjacent printed passive tools. (**B**) should show fluorescence, X-ray, MRI, and MPI image registration to anatomy. (**C**) should show a compliant tactile sensor, stiffness mapping, registration to the biopsy site, and histopathology ground truth. Use navy headings and teal, green, purple, and orange panel accents on a white background.” The authors reviewed and edited the generated output, verified the scientific content, and take full responsibility for this figure.

**Figure 4:**
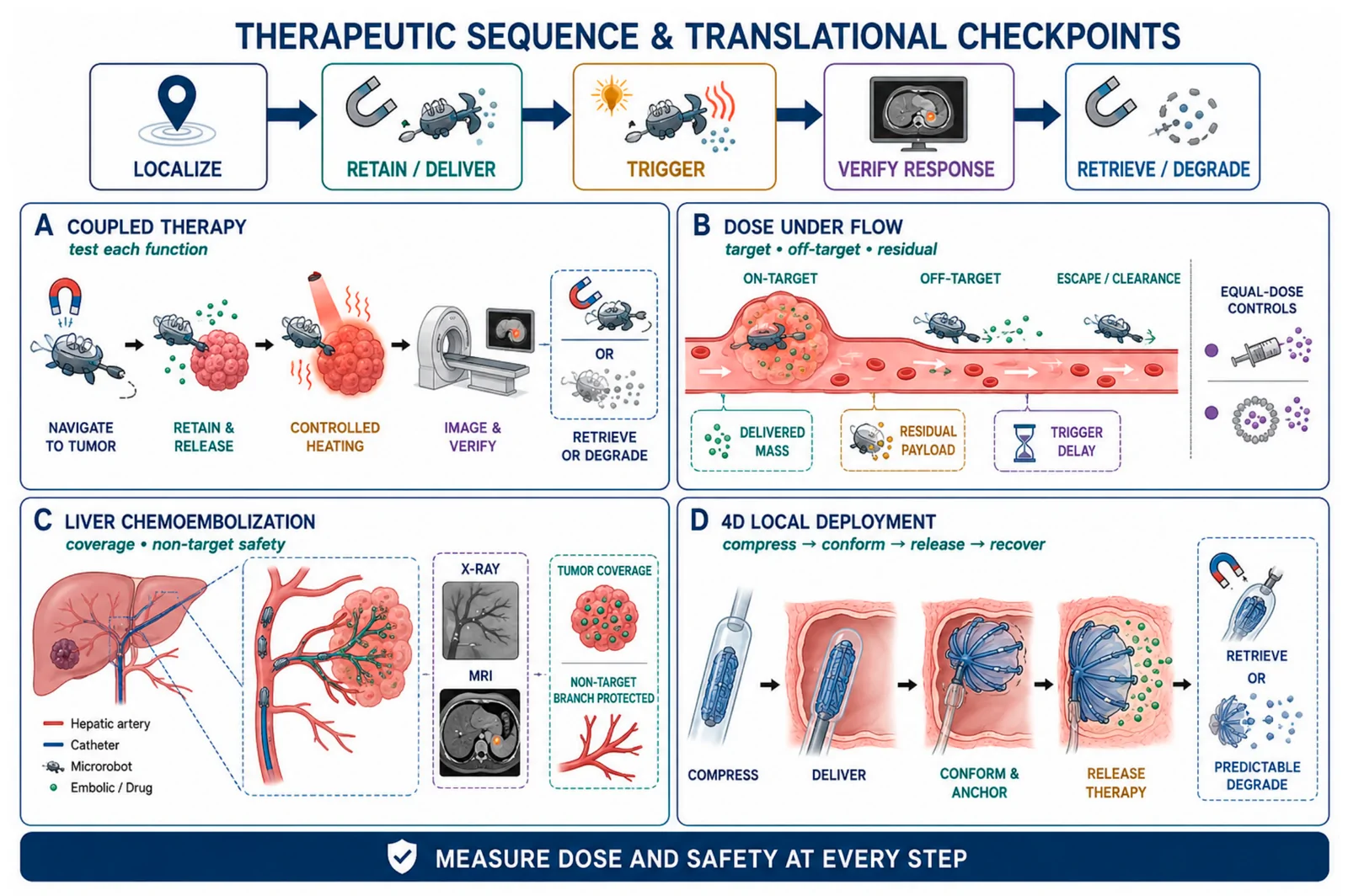
Therapeutic sequence and translational checkpoints. Navigation, local retention, trigger delivery, response verification and recovery or degradation are separate functions that require independent dose and safety measurements. Conceptual schematic; no experimental, patient or primary research data are shown. AI tool and version: OpenAI ChatGPT (version 5.6). Prompt used: “Create a four-panel scientific infographic titled Therapeutic Sequence and Translational Checkpoints. Show the overall sequence localize, retain/deliver, trigger, verify response, and retrieve/degrade. (**A**) should show coupled navigation, retention and release, controlled heating, imaging verification, and retrieval or degradation. (**B**) should depict dose under vascular flow, separating on-target delivery, off-target deposition, escape or clearance, delivered mass, residual payload, trigger delay, and equal-dose controls. (**C**) should show liver chemoembolization with catheter and microrobot delivery, X-ray/MRI verification, tumor coverage, and protection of non-target branches. (**D**) should show compression, delivery, conformal anchoring, therapy release, and retrieval or predictable degradation of a 4D local device. Add a bottom banner stating that dose and safety must be measured at every step.” The authors reviewed and edited the generated output, verified the scientific content, and take full responsibility for this figure.

**Figure 5:**
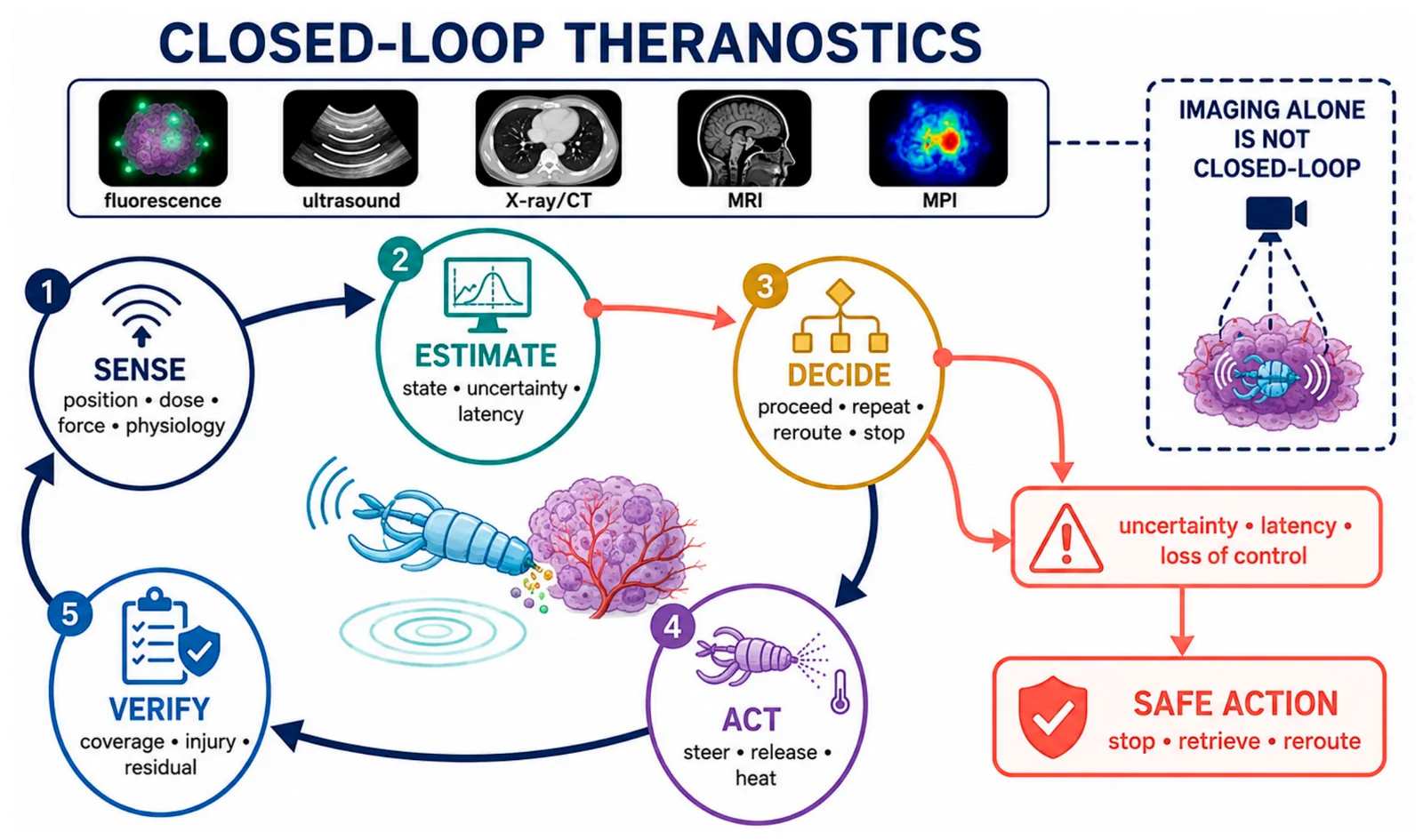
Closed-loop theranostics. Localization and physiological measurements inform state estimation, treatment decisions and actuation; uncertainty, latency and loss of control must lead to a predefined safe action. Imaging performance is system- and route-dependent. The numbered steps are 1, sense position, dose, force and physiology; 2, estimate state, uncertainty and latency; 3, decide whether to proceed, repeat, reroute or stop; 4, act by steering, releasing or heating; and 5, verify coverage, injury and residual material. Connecting arrows form the feedback cycle, and the red branch directs uncertainty, latency or loss of control to a predefined safe action. Conceptual schematic; no experimental, patient or primary research data are shown. AI tool and version: OpenAI ChatGPT (version 5.6). Prompt used: “Create a clean biomedical systems diagram titled Closed-Loop Theranostics. Place fluorescence, ultrasound, X-ray/CT, MRI, and MPI at the top. Around a nano-enabled soft robot treating a tumor, draw a numbered closed loop: 1 Sense (position, dose, force, physiology), 2 Estimate (state, uncertainty, latency), 3 Decide (proceed, repeat, reroute, stop), 4 Act (steer, release, heat), and 5 Verify (coverage, injury, residual). Add a separate dashed box explaining that imaging alone is not closed-loop, and a red safety branch in which uncertainty, latency, or loss of control leads to a safe action: stop, retrieve, or reroute. Use a white background and restrained navy, teal, gold, purple, and red accents.” The authors reviewed and edited the generated output, verified the scientific content, and take full responsibility for this figure.

**Figure 6:**
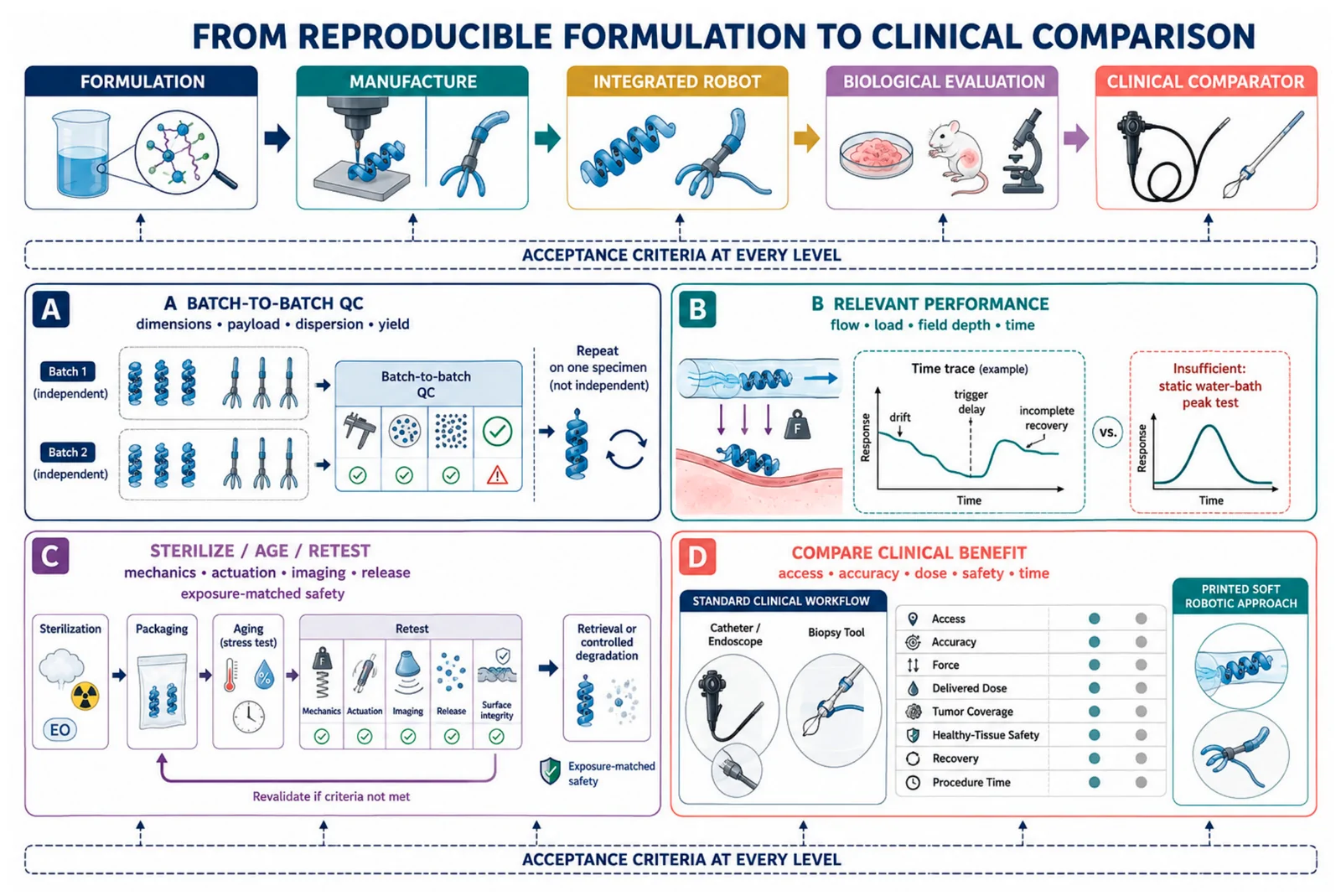
Characterization and translation pathway. Evaluation proceeds from formulation and manufacture to integrated robot performance, exposure-matched biological testing and comparison with the current clinical workflow. Arrows across the top indicate successive evaluation stages, while the dashed acceptance-criteria links connect those stages to their required checks. Within the panels, arrows indicate batch assessment, time-dependent performance, sterilization and aging followed by retesting, and comparison with the clinical workflow; the return arrow in panel C indicates revalidation when criteria are not met. Conceptual schematic; no experimental, patient or primary research data are shown. AI tool and version: OpenAI ChatGPT (version 5.6). Prompt used: “Create a publication-ready scientific infographic titled From Reproducible Formulation to Clinical Comparison. Across the top, show the pathway formulation, manufacture, integrated robot, biological evaluation, and clinical comparator, with acceptance criteria at every level. (**A**) should show independent batch-to-batch quality control for dimensions, payload, dispersion, and yield, and distinguish it from repeated measurement of one specimen. (**B**) should show performance under relevant flow, load, field depth, and time using a time-resolved response curve versus an insufficient static water-bath peak test. (**C**) should show sterilization, packaging, aging, and retesting of mechanics, actuation, imaging, release, and surface integrity, followed by retrieval or controlled degradation. (**D**) should compare the printed soft-robotic approach with standard clinical tools using access, accuracy, force, delivered dose, tumor coverage, healthy-tissue safety, recovery, and procedure time. Use four color-coded panels on a white background.” The authors reviewed and edited the generated output, verified the scientific content, and take full responsibility for this figure.

Generative AI was not used to identify, screen, select, appraise, or interpret sources or to generate the reference list. OpenAI ChatGPT (version 5.6; web application, https://chatgpt.com/, accessed on 31 August 2026) was used for limited English-language copy editing after the scientific synthesis had been completed and subsequently for revision assistance as disclosed in the Acknowledgments. OpenAI ChatGPT was used only to prepare the six conceptual schematics in Figure 1, Figure 2, Figure 3, Figure 4, Figure 5 and Figure 6; the full figure-specific prompts are disclosed in the respective captions. The authors independently reviewed and verified all AI-assisted outputs and take full responsibility for the integrity and accuracy of the manuscript.

### 1.2. Evidence Classification and Search Reproducibility

The database-specific strategies in Table 1 document the reproducible search logic used for this structured narrative review. Syntax was adapted to each database. The final revised reference set contained 87 publications: 12 core oncology-relevant printed nano-enabled soft robotic reports, 7 adjacent printed or clinical comparator reports, and 68 enabling methods, standards, or background reports.

The M/B/W/S assignments in Table 2 describe the manufacturing information, biological model, degree of workflow integration and safety endpoints reported for each study, using the definitions in Section 2 separately for each axis. An unreported endpoint is not evidence that the corresponding test failed; NR denotes unavailable reporting, whereas an S0 designation indicates that qualifying safety evidence was not identified in the report under the present framework. Performance on one axis does not justify a higher category on another. The review documents single-author screening, but does not document independent duplicate M/B/W/S classification or a formal cross-check of every assignment by both authors. The classifications should therefore be treated as descriptive author assessments without demonstrated inter-rater reliability, and borderline assignments remain a limitation of the synthesis.

## 2. Scope, Terminology, and Evidence Framework

Evidence is reported on four independent axes rather than a single Level I-IV ladder: manufacturing maturity (M0, concept only; M1, single print or batch without a reported process window; M2, repeated independent batches with a defined process window; M3, scaled and quality-controlled manufacture), biological evidence (B0, none; B1, cells or simple biofluids; B2, ex vivo tissue or anatomical phantom; B3, animal; B4, human), workflow evidence (W0, isolated function; W1, integrated bench workflow; W2, clinically realistic workflow with a comparator; W3, clinical workflow), and safety evidence (S0, none; S1, short-term in vitro; S2, exposure-matched local/systemic evaluation; S3, longitudinal biodistribution, degradation or retrieval, and device-risk evaluation). The revised review applies these axes to every core report in Table 2. NR is used where the source did not report an endpoint or did not report it in a form permitting cross-study comparison. These assignments are descriptive author assessments, not validated regulatory grades.

The restrictive core definition serves a specific analytical purpose: it isolates integrated platforms in which additive manufacture defines the device architecture, compliance and controllable actuation support robotic operation, a nanoscale phase supplies a necessary function, and that function is demonstrated in an oncology-relevant experiment. This intersection allows the review to examine whether these components work together in the same platform, rather than infer integrated capability from separate materials or robotics studies. The 12 core reports are therefore a scope-defined evidence class, not a census of cancer robotics, a measure of publication quality or a ranking above adjacent technologies. Adjacent and enabling reports remain essential for contextualizing clinically relevant functions and potential translation.

For diagnostic applications, maximum curvature, swimming speed, or workspace is secondary to whether the robot improves lesion access, localization, tissue classification, or specimen quality. Minimum outcomes include target-registration error, successful navigation, contact force, puncture or sampling success, specimen adequacy, signal-to-background ratio, imaging artifact, spatial resolution, sensitivity and specificity, and procedure time. For therapy, the corresponding outcomes are payload per robot, fraction delivered to the tumor, release kinetics under flow, energy deposition, three-dimensional thermal dose, tumor response, healthy-tissue injury, and retrieval, degradation, or clearance.

The role of printing should be stated explicitly. Printing may create an active body and magnetization pattern, a sacrificial channel or mold, a nonconductive MR Safe component, or a drug compartment. These contributions are not interchangeable. A carrier is classified as 4D only when printed architecture controls a measured, time-dependent change in shape, motion, localization, transport, release, or function. Likewise, clinical terms such as biopsy, tumor localization, targeted delivery, and therapy are used only when the corresponding function was experimentally demonstrated; otherwise, the text uses proposed, candidate, model, or adjacent comparator.

Evaluation should also consider the complete procedural system. An untethered microrobot still depends on external magnetic coils, an optical source, a scanner, an endoscope, a control model, and a recovery strategy. Device diameter, field amplitude and gradient, coil-to-target distance, imaging frame rate, localization latency, optical or acoustic attenuation, and operator workflow must therefore be reported together. The clinically relevant unit of evaluation is the robot-plus-infrastructure system rather than the printed body alone.

Application of the framework reveals a narrow evidence base. All 12 core reports demonstrate therapy or therapy-enabling transport, release or heating, whereas none reports cancer-specific diagnostic sensitivity, specificity, specimen adequacy or patient-level performance. Most core reports remain M1/B1/W1 with S0-S1 safety evidence, and no M3, B4, W3 or S3 core system was identified. This pattern indicates that therapy is more mature than diagnosis within the strict technology class, but that even therapeutic maturity is predominantly in vitro.

The strict 4D classification also reduces the apparent size of the field. Xin et al. [14] satisfied the definition through a programmed pH-driven post-print shape transformation, and the self-folded system of Darmawan et al. [21] was retained as a 4D candidate because programmed post-fabrication folding was integral to function. Thermoresponsive release without a measured architecture-encoded transformation, externally imposed movement and degradation alone were classified as responsive 3D systems rather than genuine 4D printing.

## 3. Nano-Enabled Material and Printing Design

### 3.1. Hydrogels, Biodegradable Networks, and Shape-Memory Matrices

Hydrogels are useful in this setting because their high water content, low modulus, and permeability support atraumatic tissue contact and the loading of small molecules, proteins, nanoparticles, nucleic acids, or cells. Swelling and transport can be programmed through crosslink density, ionic groups, aligned reinforcement, porosity, or bilayer architecture. For oncology, the key opportunity is to couple motion or retention with release: acidic conditions may protonate a network, proteases may cleave gelatin or peptide crosslinks, and thermoresponsive poly(N-isopropylacrylamide)-based matrices may alter volume near a designed transition temperature [23,24,25,26,27].

Printability and therapeutic transport often impose opposing requirements. Higher polymer concentration and crosslink density improve filament stability and payload retention but slow diffusion, reduce deformation, and delay degradation. Thin walls, internal porosity, microchannels, and spatially varied crosslinking are therefore more efficient than uniformly increasing responsive polymer content. Performance should be evaluated in physiological salt, serum, mucus, blood analogue, or tumor extracellular matrix because ionic strength and protein adsorption can shift swelling, diffusion, fluorescence, and surface friction.

Biodegradability alone does not establish safety. A degradable robot must retain mechanical and magnetic function during navigation and therapy, then lose integrity on a clinically appropriate timescale. Mass loss alone cannot establish this sequence. Studies should measure propulsion loss, mechanical fragmentation, particle liberation, residual payload, local concentration of degradation products, organ accumulation, and excretion. Dynamic covalent networks and shape-memory polymers provide an alternative in which a compact structure is delivered and then deployed, softened, or reconfigured; however, creep, incomplete recovery, and thermally induced tissue injury must be addressed [28,29,30,31,32] (Figure 2A).

### 3.2. Magnetic Nanocomposites

Magnetic nanocomposites are widely used in small-scale biomedical soft robots because one material family can provide torque, gradient force, MRI or MPI signal, alternating-field heating, and post-procedure retrieval. Superparamagnetic iron oxide nanoparticles reduce remanence and are commonly used as imaging or heating agents, whereas hard-magnetic particles retain programmed domains that generate complex bending, rolling, crawling, or helical propulsion. Printing and post-print magnetization can orient particles along a path or encode local magnetization vectors [33,34,35,36,37].

A central design trade-off is clear. Increasing magnetic loading improves torque, force, signal, and heating but raises viscosity, sedimentation, cure attenuation, stiffness, and the probability of particle exposure after fatigue or degradation. Aggregates create local stress concentrations and thermal hot spots. The lowest nanofiller fraction that satisfies the required actuation, imaging, and heating thresholds is therefore preferable. Reports should provide particle chemistry, size distribution, surface treatment, loading by mass and volume, dispersion method, sedimentation time, magnetic hysteresis, and evidence of retention after cycling and sterilization (Figure 2B).

Magnetic actuation, MR imaging, MPI, magnetic hyperthermia, and magnetic retrieval are distinct functions. Actuation depends on magnetic moment, field strength, gradient, torque, and workspace; MRI detects proton-signal effects and may suffer from susceptibility artifacts; MPI detects the nonlinear response of selected superparamagnetic tracers; hyperthermia depends on alternating-field frequency/amplitude and specific absorption or loss power; and retrieval depends on accessible gradients and robot integrity. A formulation optimized for one function is not automatically suitable for the others, so each field regime and endpoint must be characterized independently [38].

### 3.3. Photothermal, Conductive, and Imaging-Active Nanocomposites

Photothermal fillers used in this field include gold nanostructures, carbon nanotubes, graphene derivatives, MXenes, polydopamine, and iron-oxide-containing composites. Near-infrared irradiation can produce local heating, shape recovery, or drug release, while spatially localized printing can confine the absorber to a tumor-contacting surface or gate. Deep-tissue performance is limited by scattering, absorption, blood perfusion, and convective cooling. A therapeutic study should report wavelength, irradiance, spot size, exposure duration, beam geometry, local and surrounding tissue temperature, cooling rate, and repeated-cycle stability [39,40,41,42].

Conductive nanofillers can create strain, pressure, impedance, or temperature sensors within a compliant tip. These sensors are promising for tumor palpation and force-limited biopsy, but percolation networks are sensitive to hydration, temperature, and repeated strain. Multimaterial printing can isolate the sensing region from bending and place a compliant reference layer between the conductor and tissue. Calibration must separate temperature-induced resistance changes from mechanical strain, and electrical safety must be evaluated under wet conditions.

Fluorescent dyes, quantum dots, upconversion nanoparticles, radiopaque particles, and iron oxide can make a soft robot visible to endoscopic fluorescence, X-ray, MRI, or MPI. Imaging agents are not passive labels: they alter light scattering, cure depth, modulus, degradation, and protein adsorption. Signal should be calibrated against robot mass or number, imaging depth, motion, and orientation, and the minimum detectable intact robot or fragment should be stated. For degradable systems, fragment visibility can be as important as visualization of the intact body (Figure 2C). Table 3 summarizes the material platforms and their critical validation needs.

Melanin nanoparticles add a distinct natural-pigment platform that should not be conflated with synthetic polydopamine. Biosynthetic and natural melanin nanoparticles combine broad near-infrared absorption, photothermal conversion, photoacoustic contrast, metal-ion chelation and drug adsorption. Primary studies have demonstrated photoacoustic-guided photothermal therapy, membrane-camouflaged tumor accumulation, gene or drug co-delivery and in vivo tumor ablation [44,45,46]. These results establish cancer-nanomedicine functionality, but they do not by themselves demonstrate a printed soft robot.

A second evidence step concerns printable melanin-containing biomaterials. Melanin nanoparticle loading has enabled photothermally responsive hydrogel networks and improved light confinement and feature resolution in DLP-printed PEG-tetraacrylate/silk-fibroin hydrogels. The latter study used 0.05–0.2% (*w*/*v*) melanin in silk fibroin and reported a 2.5-fold modulus increase at the selected composite condition [47,48]. These platforms support feasibility for printable photothermal matrices, yet the final step—integration into a controllable 3D/4D-printed oncology soft robot with measured navigation, dose and safety—remains largely unexplored. Polydopamine should therefore be described as a synthetic melanin-like catecholamine polymer rather than treated as chemically identical to natural or biosynthetic melanin.

### 3.4. Printing Routes and Architecture

Direct ink writing (DIW) is well suited for millimeter- to centimeter-scale soft tools, hydrogel actuators, drug reservoirs, and multimaterial sensor-actuator systems. It tolerates high particle loading and can align fibers, mesogens, or anisotropic fillers along the print path. Printability should be documented with flow curves, yield stress, shear recovery, filament spreading, unsupported-span collapse, nozzle diameter, particle-to-nozzle size ratio, pressure, speed, layer height, and cure timing [26,49,50,51,52].

Vat photopolymerization can produce smooth surfaces, thin membranes, and complex internal chambers, whereas two-photon polymerization (2PP) produces micrometer-scale helices, porous lattices, grippers, and compartments. Their process window is controlled by resin viscosity, optical absorption and scattering, exposure dose, cure depth, shrinkage, washing, and post-curing. Nanoparticles can attenuate light and create conversion gradients; unreacted monomer and photoinitiator pose direct biological risks. Final mechanical and biological testing must use the washed and post-cured state [53,54,55,56,57].

Embedded and coaxial printing can generate freeform channels, core-shell fibers, and compartmentalized drug carriers. The decisive variables are support-bath yield stress and recovery, ink-bath diffusion, viscosity ratio, interfacial tension, core-shell flow ratio, gelation position, and support removal. Residual bath material, lumen collapse, drug cross-contamination, and shell discontinuity are relevant to oncology. Multimaterial and hybrid manufacturing can separate structural, diagnostic, therapeutic, and tissue-contacting roles; however, every additional material boundary requires adhesion, leakage, and cyclic fatigue testing [58,59,60,61,62].

Field-assisted printing and post-print magnetization make fabrication into a programming step. Magnetic fields orient particles or establish domains before curing, while the print path can encode local anisotropy and active-passive boundaries. This permits complex transformations without onboard power, but the programmed response must be evaluated at clinically relevant field depth and in viscous or confined media. A robot that moves rapidly next to a permanent magnet may not remain controllable inside the body at the working distance of a practical coil system [33,59,63,64,65] (Figure 2D).

The manufacturing strategy should be selected based on the required robotic function. DIW permits high solids, graded filaments, coaxial reservoirs, and programmed print-path anisotropy but trades resolution against nozzle pressure and particle sedimentation. Vat photopolymerization and 2PP provide finer features and internal lattices but are constrained by nanoparticle scattering, cure depth, residual monomer, and post-processing. Embedded, sacrificial, molding-assisted, and hybrid routes support channels or soft–rigid interfaces but require leakage and interface fatigue tests. Recent work on magneto-active printing, adaptive 4D structures, and nanomaterial surface modification further demonstrates that process choice changes achievable anisotropy, stimulus response, and biological interface rather than serving as a neutral shaping step [10,66,67]. Table 4 and Table 5 compare fabrication routes and stimulus-specific validation requirements.

### 3.5. Multifunction Integration and Design Trade-Offs

Multifunctionality is not additive without penalty. Raising magnetic nanoparticle content can increase torque, MPI signal and AMF heating while simultaneously increasing viscosity, optical scattering, stiffness, sedimentation and particle-release risk. Increasing photothermal absorber content can accelerate heating and release but reduce cure depth, create spatial hot spots and lower imaging or printing fidelity. Drug loading can plasticize a matrix, alter swelling and weaken interfaces, whereas higher crosslink density can restore print fidelity at the cost of diffusion, shape change and degradation. These conflicts should be reported as coupled response surfaces rather than as separately optimized demonstrations.

Device design should begin with a prioritized clinical function and a minimum sufficient material set. A formulation intended for navigation, imaging and heating should define separate acceptance thresholds for magnetic moment, localization error, thermal dose, modulus and particle retention, followed by Pareto analysis to identify compositions that satisfy all thresholds. If no common process window exists, modularization is preferable: imaging or therapeutic cargo can be confined to a replaceable compartment while the compliant actuation body uses a simpler, more reproducible formulation.

Coupled testing is essential because a multifunctional system may pass each isolated test and still fail during the combined workflow. Navigation should be repeated after drug loading and sterilization; imaging should be assessed during actuation; heating should be mapped under motion and flow; release should be quantified during and after deformation; and retrieval should be attempted after partial degradation. This sequence directly links multifunction trade-offs to the manufacturing, biological, workflow and safety axes.

## 4. 3D- and 4D-Printed Nano-Enabled Soft Robots for Cancer Diagnostics

### 4.1. Image-Compatible Lesion Access and Biopsy

Biopsy is a clinically defined procedure and is therefore used here only where sampling was demonstrated. Stormram 4 is an adjacent additively manufactured comparator, not a nano-enabled soft robot; it is a rigid serial-kinematic pneumatic system that uses printed nonconductive components. The robot has been reported as MR Safe and the needle as MR Conditional [68]. Its relevance is methodological—MR safety classification, scanner-bore workflow, target error, needle deflection, artifact, specimen adequacy, and comparison with established MR-guided breast biopsy—not evidence for a nano-enabled printed body.

Peripheral lung lesions pose a narrow, branching, mucus-lined access problem. McCandless et al. [69] demonstrated a 2.4 mm fluid-driven soft robot integrating a camera, steering, stabilization, puncture, and injection. Van Lewen et al. [70] demonstrated a millimeter-scale soft biopsy robot manufactured with molding and 3D-printed manufacturing aids. Both are labeled adjacent comparators because neither establishes a nano-enabled printed active body. Their clinically relevant endpoints are lesion access, target error, wall contact, puncture angle, sample mass, histologic adequacy, and performance under breathing, mucus, and airway collapse.

Motor-free soft robotic arms for cancer detection, microsurgery, and in situ printing further illustrate how pressure, vacuum, or stored mechanical energy can replace distal motors [71]. For diagnostic use, performance should be compared with navigational bronchoscopy, steerable needles, and concentric-tube devices rather than with unconstrained free-space motion. A highly compliant body can reduce lateral tissue force, but it can also buckle, lose orientation, and fail to generate puncture force. Compliance must therefore be co-designed with distal stiffness, force transmission, and imaging feedback (Figure 3A).

### 4.2. Imaging-Visible Navigation and Lesion Localization

Fluorescent printed hydrogels provide a material basis for optical tracking, but visibility is not tumor localization. Wang et al. [72] reported a printable fluorescent biodegradable hydrogel relevant to microrobot fabrication, whereas the Cy5 ‘intake and work’ system described by He et al. [73] is retained as an adjacent, nonprinted gastric lesion localization benchmark. Claims require signal-to-background ratio, lesion-specific binding, photobleaching, imaging depth, nonspecific adsorption, registration, and persistence after washing or tissue contact.

Magnetic particles enable deeper tracking. MRI provides anatomical context but imposes material and electromagnetic constraints. X-ray offers a high frame rate but requires radiopaque markers and exposes the patient to ionizing radiation. MPI directly detects superparamagnetic tracers without tissue background and can in principle quantify iron-oxide-containing robots, although clinical availability is limited. The imaging modality should be selected before formulation optimization because particle size, loading, and spatial distribution determine both actuation and signal. Artifact, temporal resolution, field of view, localization uncertainty, and detection during motion should be reported (Figure 3B).

### 4.3. Mechanical and Tactile Diagnosis

Many tumors differ mechanically from surrounding tissue, creating an opportunity for printed soft robotic palpation. A compliant distal tip can combine controlled indentation with ionic, resistive, capacitive, or magnetic sensing. Multimaterial printing can isolate the sensor from bending strain, place a tissue-matched contact layer over the transducer, and create a reference structure that reduces dependence on approach angle. The useful output is a spatially registered stiffness or force map, not an uncalibrated resistance trace [27,36,74,75,76].

Cancer-specific evidence remains limited. Generic soft sensors are frequently tested on idealized objects, while tumor phantoms may omit capsule thickness, lesion depth, viscoelasticity, and surrounding heterogeneity. A credible study should use layered phantoms and fresh tissue with histopathologic ground truth, report depth sensitivity and spatial resolution, and quantify confounding from hydration, curvature, robot orientation, and normal-force variation. Sensitivity and specificity should be calculated at the lesion level, and a suspicious tactile region must be registered to imaging so that it can be biopsied or treated (Figure 3C). Table 6 summarizes representative diagnostic systems, scope classifications, and evidence gaps.

### 4.4. Relative Maturity of Diagnostic Evidence

Within the strict core evidence set, no study demonstrated cancer diagnosis using a biological ground truth, together with sensitivity, specificity, classification performance or specimen adequacy. Fluorescence, MPI visibility, tactile response and successful navigation were therefore classified as enabling measurements rather than indicators of cancer diagnosis. Stormram 4, peripheral-lung biopsy robots and Cy5 localization remain clinically informative adjacent comparators because they define workflow endpoints, but they do not satisfy the complete nano-enabled printed soft-robot definition.

This asymmetry is a principal finding rather than a gap to be filled by conceptual combinations. Therapeutic evidence includes cell-level drug release, hyperthermia and combination treatment effects, whereas diagnostic evidence has not yet reached cancer-specific accuracy testing in the core technology class. Future diagnostic studies should preregister a lesion-level ground truth and report false-positive and false-negative results, localization error, specimen adequacy and comparison with the current biopsy or imaging workflow.

## 5. 3D- and 4D-Printed Nano-Enabled Soft Robots for Cancer Therapy

### 5.1. Targeted Delivery and Local Retention

The therapeutic rationale for a microrobot is straightforward: to transport active cargo against diffusion and bulk flow, retain it near a lesion, and release it only after localization. Biodegradable printed microswimmers and helical robots have demonstrated magnetic navigation, cargo loading, and enzymatic degradation [12,13]. An environmentally adaptive microcrab changed shape and delivered particles for localized cancer cell treatment [14]. These studies establish that geometry, magnetic response, targeting chemistry, degradation, and release can be encoded in one printed structure, but the delivered dose per robot and the fraction of an administered population reaching the tumor remain central quantitative gaps.

Magnetically actuated, degradable microrobots have combined drug release with alternating-field hyperthermia [15], while porous magnetic robots have used ultrasound to accelerate release [16]. Self-rolled robots have integrated magnetic guidance, real-time X-ray visualization, photothermal release, and retrieval [17]. Helical systems have added magnetic nanoparticle retrieval and sequential dual-drug release [18,19]. Each function is individually attractive, but integration increases formulation complexity, field requirements, and failure modes. Release, retrieval, imaging, and heating should therefore be validated both separately and as a coupled workflow (Figure 4A).

### 5.2. Pharmaceutical Formulation, Stability, and Dose Accounting

For drug-bearing robots, formulation performance must be reported independently of locomotion. Minimum attributes include drug identity and physical state, loading capacity, encapsulation efficiency, content uniformity per robot and per batch, free-drug carryover, pre-trigger leakage, storage and in-use stability, sterilization compatibility, release kinetics in sink and physiologically relevant flow conditions, and residual drug after retrieval or degradation. Robot count or administered suspension volume is not a dose unless linked to drug mass and exposure.

Pharmacological evaluation should compare equal total drug doses delivered as free drug, nonrobotic carrier, unloaded robot, triggered robot, and robot plus energy without drug. Concentration–time profiles at the target and off-target compartments, pharmacokinetics, biodistribution, therapeutic index, and mechanism-appropriate biomarkers are needed before “targeted delivery” or combination benefit can be claimed. The AI-guided 4D-printed microneedle platform reported by Lee et al. [77] is included as an adjacent printed therapeutic platform illustrating how geometry, drug delivery, and biological performance can be co-optimized, although it is not an oncology soft robot.

### 5.3. Stimulus-Triggered Release and Combination Therapy

Four-dimensional behavior may improve tumor contact, expose a shielded drug surface, or open a compartment after localization, but trigger feasibility is route- and tissue-dependent. Tumor pH and enzyme activity are heterogeneous and may not provide a sharp or sufficiently rapid switch; light requires superficial, surgical, endoscopic, or fiber access; ultrasound requires defined pressure, frequency, attenuation, and cavitation limits; and thermal activation must be separated from unintended tissue heating. The PNAGA study reported greater release at 45 °C than at 25 °C [20]; 45 °C is above normal physiological temperature and therefore requires explicit thermal-dose and healthy-tissue safety evaluation rather than being treated as intrinsically physiological. Table 5 summarizes validation requirements.

Cumulative release in a vial is insufficient. The relevant measurement is release at the target under flow, including the delay between triggering and therapeutic concentration, the fraction released outside the lesion, and the residual dose after retrieval or degradation. For sequential systems, the first trigger must not prematurely release the second agent. Combination benefit should be demonstrated against each single modality with the same total drug and robot burden. Heat, acoustic pressure, and drug concentration are coupled dose variables and should be reported together (Figure 4B).

### 5.4. Photothermal Therapy, Magnetic Hyperthermia, and Chemoembolization

Local heating is a natural match for printed nano-enabled robots because navigation can precede energy delivery. Photothermal therapy is most realistic for superficial lesions, endoscopically accessible cavities, surgical fields, or tissues reached by a fiber, where light delivery and temperature monitoring are feasible. Magnetic hyperthermia avoids optical penetration limits but requires a practical alternating-field coil and sufficient magnetic loss within the robot. TriMag microrobots integrate magnetic actuation, MPI, and hyperthermia, demonstrating a direct theranostic architecture [11].

Imaging, propulsion, and heating do not necessarily share the same optimal particle properties or field regime. Increasing iron-oxide loading may improve MPI signal and heating while reducing print resolution, flexibility, and degradation. The navigation field, imaging sequence, and hyperthermia field should be independently characterized to prevent unintended heating during motion or loss of control during treatment. A high robot-surface temperature does not establish adequate tumor coverage; studies should map three-dimensional temperature under flow or perfusion and report duration above therapeutic thresholds.

Thermal efficacy and safety should be reported as time–temperature histories and three-dimensional dose maps, not only peak temperature. A commonly used summary is cumulative equivalent minutes at 43 °C (CEM43): CEM43 = Σ Δt·R^(43 − T), where R is conventionally 0.5 at T ≥ 43 °C and 0.25 at T < 43 °C, with assumptions stated [78]. CEM43 should be mapped in tumor and adjacent tissue under flow or perfusion, together with probe or imaging uncertainty, cool-down, repeated exposures, and thermal controls. TriMag should be described as a 3D-printed platform for magnetic actuation, MPI, and hyperthermia; MRI should not be inferred without separate validation [11].

For vascular tumors, an adjacent nonprinted system-level example is a multifunctional microrobot visualized by real-time X-ray and MRI for liver-cancer chemoembolization [79]. This work illustrates how robotic delivery can be integrated into an established interventional pathway. Future comparisons should quantify embolization distribution, tumor coverage, non-target vessel occlusion, contrast dose, catheter compatibility, and whether magnetic guidance improves efficacy or safety relative to conventional transarterial delivery (Figure 4C).

### 5.5. Tethered Focal Injection and Local Deployment

The peripheral-lung soft robot provides a clinically intuitive sequence: reach a suspicious lesion, confirm position, puncture, and inject a local agent through the same device [69]. A tethered architecture avoids the most difficult clearance problem faced by free microrobots and can accommodate chemotherapy, ablation agents, radionuclide carriers, hydrogels, or immunomodulators. The design challenge is to prevent cross-contamination between diagnostic and therapeutic channels and to verify that injection pressure does not displace the tip or cause extravasation.

Deployable 4D-printed tools may also anchor within a resection cavity or hollow organ, conform to an irregular tumor margin, and release therapy over a defined period. Shape-memory and swelling-driven devices can be compressed for insertion and expanded at the target. Although many such studies remain material demonstrations, the oncology opportunity includes postoperative local chemotherapy, intracavitary therapy, and temporary brachytherapy-compatible carriers. The device should be retrievable or degrade predictably after the therapeutic window [28,32,54,57,80] (Figure 4D). Table 7 summarizes representative therapeutic systems and their priority translational requirements.

## 6. Integrated Theranostics, Imaging, and Closed-Loop Control

Theranostics is more than co-loading an imaging agent and a drug. A closed-loop system must localize the robot, estimate its relationship to the tumor and surrounding anatomy, deliver or trigger therapy, and verify distribution or response. Fluorescence, endoscopic vision, X-ray, ultrasound, MRI, and MPI provide different combinations of depth, frame rate, tissue contrast, and direct robot detection. Multimodal systems are justified when one modality provides anatomy and another quantifies the robot, but added markers and hardware should produce a measurable improvement in target error, dose, or safety.

Control models calibrated in water may fail in blood, mucus, bile, or confined tissue cavities. Flow, wall contact, friction, deformation, magnetic-field nonuniformity, and payload loss all change the relationship between command and displacement. Imaging latency and localization uncertainty should be incorporated into controller evaluation rather than treated as post hoc technical details. For tethered systems, tip tracking and force sensing may provide a safer near-term route than fully autonomous untethered control.

The most practical architecture may be modular. A printed soft body can provide steering and tissue contact, a replaceable cartridge can carry the drug, a thin region can contain the imaging agent, and an external system can supply energy. Modular design separates high-risk therapeutic materials from reusable actuation hardware, facilitates sterilization, and permits independent validation of the payload, robot, imaging marker, and controller. It also enables a clean boundary between diagnostic and therapeutic channels after biopsy.

Closed-loop treatment requires quantitative data streams. Localization should include uncertainty and latency; therapeutic delivery should include mass, concentration, temperature, or acoustic energy; mechanical state should include strain, force, and wall contact; and biological response should include perfusion, contrast change, tumor coverage, or tissue injury. Safety logic must specify when to stop, retrieve, re-route, or repeat treatment. A system that cannot recognize loss of control is not closed-loop, even if imaging is available (Figure 5). Table 8 compares the principal imaging modalities and their system-dependent limitations.

## 7. Characterization, Safety, and Translation

### 7.1. Printing and Nanocomposite Reproducibility

A printed nano-enabled robot should be characterized as a hierarchy: formulation, printed material, internal interface, robot, and clinical task. Formulation data include polymer composition, nanomaterial chemistry, size and loading, surface treatment, dispersion method, rheology or cure kinetics, optical attenuation, pot life, storage stability, and sedimentation. Printed material data include dimensions, voids, conversion, anisotropy, modulus, toughness, swelling, conductivity, magnetic response, thermal conversion, and environmental dependence. Interfaces require peel, interlayer fracture, burst, leakage, or particle retention tests selected for the architecture [52,62,81,82,83].

A successful image is not evidence of manufacturing reproducibility. Independent batches, print yield, dimensional variation, payload variation, nanofiller distribution, magnetic moment, imaging signal, heating rate, and acceptance criteria should be reported. For a microhelix, a small error in diameter or pitch can change propulsion and dose. For a soft biopsy tool, wall thickness and channel geometry influence bending, force, leakage, and burst pressure. Between-batch variability should be analyzed separately from repeat measurements on one specimen (Figure 6A).

For translation, manufacturing reproducibility should also be evaluated together with sterilization and storage. Independent production lots should be compared using predefined acceptance limits for dimensions, mechanical response, nanoparticle loading and retention, degradation profile and the intended actuation, imaging or release endpoint. Variation introduced by printing, washing, curing and sterilization should be distinguished from repeated measurement of one specimen. Such testing would establish whether the final processed device retains its specified function across lots; the current core evidence set does not by itself establish this level of reproducibility.

### 7.2. Oncology-Specific Performance Metrics

Diagnostic studies should report target error in an anatomical coordinate system, navigation success, contact force, puncture success, specimen volume and quality, imaging artifact, localization latency, and time to completion. A stiffness-sensing device should report spatial resolution, depth sensitivity, repeatability, and classification performance with histopathologic or well-characterized ground truth. Therapeutic studies should report robot number or mass, payload per robot, total administered dose, target delivery fraction, concentration-time profile, energy input, three-dimensional thermal map, tumor response, and injury to adjacent tissue.

Maximum swimming speed, bending angle, or equilibrium release is useful but cannot substitute for application outcomes. Time-resolved curves reveal drift, overshoot, incomplete recovery, degradation, and trigger delay. Performance should be measured under load and in relevant media. Magnetic studies require field amplitude, gradient, frequency, coil geometry, coil-to-target distance, workspace, and medium viscosity. Optical and acoustic studies require beam or transducer geometry, intensity, duration, attenuation, and tissue-equivalent conditions (Figure 6B).

### 7.3. Retrieval, Degradation, Biological Evaluation, and Sterilization

Retrieval is preferable when a robot contains persistent nanoparticles, high drug loading, sharp fragments, or uncertain degradation products. A retrieval claim should include success rate, time, dependence on robot orientation, recovery after partial damage, and residual material. For degradable robots, the desired sequence is function first, therapy second, and safe loss of integrity last. Studies should establish loss of propulsion, fragmentation size, drug and nanoparticle release, biodistribution, organ accumulation, and excretion.

Biological evaluation should match exposure. A short-term luminal robot requires cytocompatibility, extractables, hemocompatibility when relevant, mechanical integrity, and sterilization compatibility. An intravascular or implantable microrobot requires evaluation of thrombosis, immune response, endothelial injury, organ accumulation, and degradation products. A tumor-cell viability assay demonstrates biological activity but not a therapeutic index. Tumor-bearing animal studies should include healthy-tissue histology and a control receiving the same drug or energy without robotic localization.

Study-level safety evidence is summarized in Table 2 rather than inferred from functionality. Across the 12 core reports, short-term cytocompatibility or cancer cell response was common, but exposure-matched healthy-tissue toxicity, hemocompatibility, immune response, biodistribution, nanoparticle clearance, degradation-product analysis and longitudinal in vivo safety were generally NR. Retrieval was demonstrated in selected systems [17,18,19], but complete mass balance after partial damage was not established. No core report reached S3 under the present framework. The resulting gap between functional demonstration and safety validation is therefore a central conclusion of this review, not a generic caveat.

Sterilization is an interfacial and nanocomposite stress test. Ethylene oxide, radiation, moist heat, ultraviolet exposure, alcohol and chemical disinfectants can change crosslink density, swelling, adhesion, conductivity, magnetization, fluorescence, drug stability, residual monomer and nanoparticle retention. Sterilization compatibility should be established on the final packaged device by repeating key mechanical, actuation, imaging, release and particle-shedding tests after processing and aging. ISO 11135 and ISO 11137-1 provide process requirements for ethylene oxide and radiation sterilization, respectively, but they do not establish that a particular nanocomposite retains clinical function after treatment [84,85]. Biological evaluation should follow the intended contact type and duration within a risk-management process, consistent with ISO 10993-1:2025 and ISO 14971:2019 [86,87].

Sterilization efficacy and preservation of device function are separate validation questions. Cleaning, disinfection and laboratory ultraviolet exposure should not be assumed to constitute a validated terminal sterilization process. For each candidate material–device combination, matched pre- and post-processing measurements should assess mechanical behavior, interfacial integrity, nanoparticle retention or shedding, degradation kinetics and the intended diagnostic or therapeutic function, including after storage. These are proposed validation priorities, not outcomes demonstrated across the reviewed core systems. A compatible process must therefore be selected and validated for the final device rather than inferred from the polymer or nanofiller alone [84,85,86,87].

### 7.4. Clinical Positioning and Regulatory Strategy

Demonstrated readiness, translationally relevant adjacent evidence and author-proposed clinical opportunities must be separated. Demonstrated core evidence currently consists mainly of in vitro transport, release, heating and retrieval workflows. Adjacent systems demonstrate clinically relevant access, imaging or biopsy methods without satisfying the complete core definition. On this basis, the authors propose—but do not claim as established advantage—that tethered, retrievable systems used through established access routes may offer the nearest-term opportunities in MRI-guided breast biopsy, bronchoscopy, gastrointestinal endoscopy, catheter-based liver treatment, surgical cavities and resection beds. Untethered vascular or interstitial systems remain prospective because scalable field generation, quantitative tracking, swarm-dose accounting and verified clearance have not been demonstrated together.

Clinical benefit must be tested against the current standard of care. Reaching smaller airways, reducing tissue force, improving localization or biopsy yield, concentrating drug, reducing systemic exposure and enabling single-session theranostics are hypotheses to be evaluated, not established advantages of the technology class. Table 9 therefore labels robotic value as author proposed and pairs each hypothesis with comparative endpoints. Translation will depend more on reproducible evidence, a recoverable failure mode and a clear use case than on increasingly complex demonstrations.

Diagnostic and therapeutic maturity should be interpreted separately. Within the predefined search and eligibility framework, the core reports demonstrate treatment-related functions or therapy-enabling transport, release and heating, predominantly in vitro. This is evidence of functional feasibility, not established therapeutic efficacy in patients. By contrast, making a robot visible or tracking its position does not establish cancer detection, lesion discrimination or diagnostic accuracy. Diagnostic maturity requires cancer-specific endpoints evaluated against an appropriate reference standard, such as sensitivity and specificity or specimen adequacy with histopathological confirmation. Thus, the relative lead of therapeutic demonstrations does not imply clinical readiness, and diagnostic performance remains largely unvalidated within this core evidence set.

### 7.5. Scalability, Durability, Energy, and Clinical Implementation

Scale-up requires controlled raw material specifications, nanoparticle dispersion, print yield, dimensional and payload uniformity, post-cure/wash validation, sterile packaging, shelf life, and lot-release criteria. Durability testing should reproduce storage, deployment, cyclic actuation, abrasion, field exposure, and contact with blood, mucus, bile, or tissue. Persistent function must be separated from safe end-of-life behavior: fatigue must not liberate particles or drugs before retrieval or planned degradation.

Power and energy belong to the complete system. Reports should include coil or magnet geometry, electrical power, cooling, duty cycle, acoustic or optical source efficiency, scanner compatibility, workspace, and maximum operator/patient exposure. Thermal management must cover the robot, target, adjacent tissue, actuator hardware, and cables/coils. Clinical implementation further requires failure recovery, sterility, setup time, user training, human factors testing, cybersecurity/software validation where applicable, and a regulatory strategy for the combined device, drug, imaging agent, and energy source. Table 9 and Table 10 translate these requirements into clinical comparators and a minimum reporting framework.

## 8. Conclusions

The strict evidence synthesis identified 12 experimentally demonstrated core systems, all directed toward therapy or therapy-enabling transport, release, heating or theranostics. No core report established cancer-specific diagnostic sensitivity, specificity or specimen adequacy, and no human study was identified. Most systems remained at M1/B1/W1 with S0-S1 safety evidence: a single-platform manufacturing demonstration, cell or simple-fluid biology and an integrated bench workflow, with safety either unreported or limited to short-term in vitro assessment. Therapy is therefore more mature than diagnosis within this technology class, but neither has reached clinical validation. This comparison is restricted to the predefined search and eligibility framework: no human validation or qualifying cancer-specific diagnostic performance was identified within that framework. The stronger representation of therapeutic functions reflects preclinical feasibility demonstrations rather than a validated clinical treatment. Imaging visibility and navigation should therefore remain distinct from diagnostic validation, just as transport, release or heating should remain distinct from patient-level therapeutic benefit.

Only one system clearly met the strict 4D definition through a programmed post-print transformation, with one additional self-folding system retained as a 4D candidate. The most frequent missing steps were independent-batch reproducibility, clinically meaningful delivered-dose accounting, exposure-matched healthy-tissue assessment and long-term fate. Retrieval was reported selectively, whereas complete biodistribution, nanoparticle clearance, sterilization compatibility and longitudinal safety were rarely or not reported. These omissions prevent visually compelling motion, imaging and release demonstrations from being interpreted as translational readiness.

The field should therefore prioritize a smaller number of integrated, recoverable systems with explicit process windows, quantitative localization and therapeutic endpoints, and risk-based safety programs. The M/B/W/S tuple is useful as a transparent reporting and comparison tool, but it is not regulatory grade. Progress should be measured by movement from M1/B1/W1/S0-S1 toward repeated manufacture, clinically realistic comparators, in vivo benefit and verified retrieval or clearance, rather than by the number of functions incorporated into one device.

## 9. Future Perspective

The strongest near-term opportunities are candidate systems that combine soft compliance with a defined recovery route and established imaging. Stormram 4 and peripheral-lung robots are instructive adjacent comparators rather than nano-enabled printed exemplars; they demonstrate how clinical access, MR safety, sampling, and retrieval can be framed. Untethered printed microrobots offer possibilities for localized delivery and hyperthermia, but most evidence remains preclinical and requires dose accounting, image-based control, practical field systems, exposure-matched safety, and verified clearance before clinical benefit can be inferred.

Further progress will depend less on adding modalities and more on demonstrating comparable evidence. Process windows, nanofiller dispersion, interfacial adhesion, target error, specimen quality, delivered dose, three-dimensional thermal history, retrieval or degradation, sterilization, and application-level benefit should become routine reporting elements. With these controls, printed nano-enabled soft robots can evolve from visually impressive prototypes into reproducible platforms for cancer diagnosis, focal therapy, and single-session theranostics.

## Figures and Tables

**Table 1:** Database-specific literature search strategies.

Database	Coverage and Date	Search Strategy	Selection Note
PubMed	1 January 2000–31 August 2026; final search 31 August 2026	((“3D print*” OR “4D print*” OR “direct ink writing” OR stereolithography OR “digital light processing” OR “two-photon polymerization”) AND (“soft robot*” OR microrobot* OR microswimmer*) AND (nanoparticle* OR nanocomposite* OR “nano-enabled”) AND (cancer OR tumor OR biopsy OR diagnosis OR imaging OR “drug delivery” OR hyperthermia OR chemoembolization OR theranostic*))	Title/abstract screening followed by full-text scope classification.
Web of Science Core Collection	1 January 2000–31 August 2026; final search 31 August 2026	TS = ((3D print* OR 4D print* OR direct ink writing OR stereolithography OR digital light processing OR two-photon polymerization) AND (soft robot* OR microrobot* OR microswimmer*) AND (nanoparticle* OR nanocomposite* OR nano-enabled) AND (cancer OR tumor OR biopsy OR diagnosis OR imaging OR drug delivery OR hyperthermia OR chemoembolization OR theranostic*))	Topic search; cited-reference and citing-article searches applied.
Scopus	1 January 2000–31 August 2026; final search 31 August 2026	TITLE-ABS-KEY((3D W/1 print* OR 4D W/1 print* OR “direct ink writing” OR stereolithography OR “digital light processing” OR “two-photon polymerization”) AND (“soft robot*” OR microrobot* OR microswimmer*) AND (nanoparticle* OR nanocomposite* OR “nano-enabled”) AND (cancer OR tumor OR biopsy OR diagnosis OR imaging OR “drug delivery” OR hyperthermia OR chemoembolization OR theranostic*))	Title/abstract/keyword search; duplicates reconciled against DOI and title.
Google Scholar	1 January 2000–31 August 2026; final search 31 August 2026	Phrase combinations of printing route + soft robot/microrobot + nanomaterial + cancer function; examples: “3D printed” microrobot cancer nanoparticle; “4D printed” soft robot tumor drug delivery; two-photon polymerization microswimmer cancer hyperthermia.	Used for citation chaining and papers not consistently indexed; not used alone to establish eligibility.

Note: Core evidence required an additively manufactured compliant robot body, a functionally necessary nanoscale phase, robotic control or actuation, and an experimentally demonstrated oncology-relevant function. Adjacent evidence met only part of this definition. Enabling evidence supported materials, printing, imaging, safety, standards, or clinical comparators without demonstrating the complete integrated system. In the database search expressions, an asterisk (*) denotes a truncation wildcard used to retrieve alternative word endings; it is not a statistical-significance or footnote symbol.

**Table 2:** Core evidence set: experimentally demonstrated printed nano-enabled soft robotic systems with oncology-relevant functions.

Study and Strict Classification	Printed Platform, Size and Nano-Enabled Function	Oncology Function and Model	Quantitative or Imaging Evidence Reported	M/B/W/S Assessment, Safety Evidence and Principal Limitation
TriMag microrobot [11] 3D printed; not genuine 4D	2PP hydrogel microrobot; microscale. Three magnetic phases support actuation, MPI visibility and AMF heating.	Integrated theranostic architecture; oncology-relevant hyperthermia, without cancer-specific diagnosis.	Magnetic locomotion, MPI signal and AMF heating reported; cancer diagnostic sensitivity/specificity NR.	M1/B1/W1/S1. In vitro functional and short-term compatibility evidence; in vivo localization, thermal coverage, clearance and long-term particle fate remain NR.
Ceylan et al. [12] 3D printed; not genuine 4D	2PP biodegradable double-helical microswimmer with magnetic nanoparticles and functional cargo; microscale.	Cargo delivery, cell targeting and enzymatic degradation in microfluidic/in vitro models.	Magnetic navigation, cargo loading/release and degradation measured; dose reaching a tumor, biodistribution and retrieval NR.	M1/B1/W1/S1. Cytocompatibility/degradation demonstrated; batch reproducibility, in vivo clearance and long-term safety NR.
Wang et al. [13] 3D printed; not genuine 4D	2PP soft helical microswimmer with magnetic nanoparticles and enzyme-cleavable matrix; microscale.	Biodegradable navigation and cargo transport; enabling therapeutic platform rather than cancer-specific diagnosis.	Propulsion and enzymatic degradation reported; tumor delivery fraction, administered dose and healthy-tissue effect NR.	M1/B1/W1/S1. In vitro degradation evidence; particle liberation, organ accumulation and longitudinal safety NR.
Xin et al. [14] Genuine 4D: programmed post-print pH-driven shape morphing	2PP hydrogel microcrab/fish with magnetic nanoparticle actuation; programmed differential swelling; microscale.	Microparticle handling and doxorubicin release to HeLa cells in an artificial vascular model.	pH-dependent transformation, transport/release and cancer-cell response reported; in vivo trigger selectivity and systemic dose NR.	M1/B1/W1/S0. Cancer cell efficacy was reported, but a separate safety assessment was NR; tumor pH heterogeneity, off-target release, biodistribution and clearance remain unresolved.
Park et al. [15] 3D printed responsive system; not strict 4D	2PP helical PEGDA/PETA robot containing Fe_3_O_4_ and 5-FU; microscale.	Magnetic navigation, AMF-controlled drug release and hyperthermia in cancer cell assays.	Normal, burst and constant release modes and cell-viability reduction reported; 3D thermal dose and in vivo tumor response NR.	M1/B1/W1/S0. Cancer cell activity is not a safety test; AMF depth, healthy-tissue thermal exposure, nanoparticle fate and sterilization NR.
Park et al. [16] 3D printed responsive system; not strict 4D	3D porous magnetic helical microrobot with degradable polymer and drug cargo; microscale.	Magnetic targeting with ultrasound-accelerated drug release in vitro.	Release acceleration and targeted cell response reported; tissue acoustic dose, cavitation margin and in vivo delivery fraction NR.	M1/B1/W1/S0. Therapeutic cell response was reported, but exposure-matched safety, premature release, degradation products and longitudinal safety were NR.
Nguyen et al. [17] 3D printed/self-rolled; 4D status not established under strict definition	Magnetically guided self-rolled microrobot with radiopaque/imaging and photothermal components; microscale.	Targeted delivery, real-time X-ray visualization, NIR-triggered release and magnetic retrieval.	Integrated navigation, X-ray tracking, release and retrieval reported; retrieval failure rate after damage and residual mass NR.	M1/B1/W1/S1. Retrieval demonstrated; vessel injury, retained fragments, biodistribution and repeated-use safety NR.
Lee et al. [18] 3D printed; not genuine 4D	Magnetic helical drug-delivery microrobot with nanoparticle retrieval function; microscale.	Magnetic navigation, drug delivery and magnetic nanoparticle recovery in vitro.	Locomotion, release and nanoparticle retrieval reported; tumor delivery fraction and in vivo therapeutic index NR.	M1/B1/W1/S0. Therapeutic cell evidence was reported; residual nanoparticles, organ fate and long-term safety assessment NR.
Lee and Park [19] 3D printed responsive system; not strict 4D	Magnetic helical microrobot carrying sequential dual-drug payloads; microscale.	Staged combination drug release and nanoparticle retrieval in vitro.	Sequential release and cell response reported; trigger cross-talk, dose equivalence and combination-index analysis incompletely reported.	M1/B1/W1/S0. Combination efficacy was reported without a separate exposure-matched safety program; in vivo pharmacokinetics, retrieval completeness and healthy-tissue toxicity NR.
Zhou et al. [20] Stimulus-responsive 3D print; strict 4D transformation not demonstrated	3D-printed PNAGA thermosensitive hydrogel microrobot with anticancer drug.	Temperature-triggered release and cancer cell treatment in vitro.	Greater release at 45 °C than 25 °C reported; 45 °C exceeds physiological temperature. CEM43 and perfused-tissue dose NR.	M1/B1/W1/S1. Cell-level effect; deep-tissue triggering, healthy-tissue thermal injury and degradation fate unresolved.
Darmawan et al. [21] 4D candidate: programmed self-folding	Self-folded magnetic microrobot with drug compartment and ultrasound-responsive release; microscale.	Active delivery and rapid ultrasound-triggered drug release in vitro.	Folding and release kinetics reported; tissue attenuation, cavitation threshold and in vivo delivery fraction NR.	M1/B1/W1/S0. Therapeutic activity was reported; separate safety evidence, repeatability of programmed transformation, degradation products and long-term safety NR.
Li et al. [22] 3D printed multidrug system; not genuine 4D	Magnetically guided printed microrobot carrying multiple therapeutic agents; microscale.	Synergistic combination treatment in cancer models.	Multidrug release and treatment response reported; harmonized per-robot dose, trigger orthogonality and full pharmacokinetics NR.	M1/B1/W1/S0. Preclinical/cell-level efficacy was reported; batch dose uniformity, biodistribution, retrieval and chronic safety NR.

Note: Classifications are the authors’ structured assessment of the 12 application-focused core reports. NR, not reported or not reported in a form permitting cross-study comparison. M/B/W/S definitions are provided in Section 2. The table deliberately separates demonstrated endpoints from missing evidence and does not treat imaging visibility as cancer diagnosis.

**Table 3:** Nano-enabled material platforms for cancer-diagnostic and cancer-therapeutic printed soft robots.

Material Platform	Nanoscale Functional Phase	Typical Printing Route	Oncology Function	Critical Validation
Hydrogel/GelMA/PEGDA nanocomposite	SPION, Au, PDA, MXene, fluorescent agents	DIW, DLP/SLA, 2PP, embedded	Soft contact; swelling; drug reservoir; imaging	Physiological-media response; payload leakage; particle retention; degradation products
Shape-memory polymer/dynamic network	Fe_3_O_4_, carbon, Au/PDA photothermal phase	DLP/SLA, 2PP, multimaterial 4D printing	Compact delivery; deployment; thermally gated exposure or retrieval	Activation threshold; creep; recovery under load; local temperature
Magnetoactive elastomer or hydrogel	SPION or hard-magnetic micro/nanoparticles	Field-assisted DIW/DLP; post-print magnetization; 2PP	Navigation; anchoring; MRI/MPI; AMF hyperthermia	Sedimentation; stiffness; field scalability; filler release
Photothermal nanocomposite	Au nanorods, PDA, CNT, graphene, MXene	DIW; localized DLP/2PP; coating	NIR-triggered shape change; release; photothermal therapy	Penetration; hot spots; perfusion cooling; photostability
Conductive/ionic composite	CNT, graphene, AgNW, MXene, conducting polymers	Multimaterial DIW; embedded conductors	Tactile mapping; force feedback; Joule heating	Wet drift; percolation fracture; cross-talk; insulation
Imaging-active composite	SPION, QD, UCNP, fluorescent dye, radiopaque nanoparticle	2PP/DLP; coating; compartment loading	Fluorescence, X-ray, MRI, MPI, multimodal tracking	Signal calibration; artifact; photobleaching; marker retention
Adjacent liquid-metal/hybrid circuit	Ga–In droplets or channels plus nanofiller skin	Embedded printing; channel filling; hybrid assembly	Stretchable heater, electrode, sensor, connector	Leakage; oxide instability; puncture and sterilization resistance

Note: Author synthesis based on representative material, process and function reports [6,7,9,43].

**Table 4:** Comparative manufacturing strategies for nano-enabled soft robotic and responsive biomedical platforms.

Fabrication	Nanocomposite/Material	Actuation or 4D Response	Imaging	Advantages	Limitations	Representative Application
DIW/extrusion	Hydrogel, elastomer, SPION, CNT/MXene, drug-loaded inks	Pneumatic, magnetic, swelling, thermal	Optical, MRI/MPI, X-ray markers	High loading; multimaterial; channels	Moderate resolution; shear; sedimentation	Soft tools, reservoirs, sensors
DLP/SLA	Photocurable hydrogel, SMP, magnetic/photothermal resin	Shape memory, swelling, magnetic	Optical, MRI, CT marker	Complex cavities; smooth surfaces	Scattering; residuals; limited cell/drug compatibility	Deployable tools, grippers, carriers
2PP	Photopolymer with magnetic or optical nanoparticles	Magnetic swimming, shape change	Fluorescence, X-ray/MRI marker	Micrometer features; lattices	Low throughput; small payload; stringent chemistry	Helices, microswimmers, microgrippers
Embedded/sacrificial	Soft ink in yield-stress bath; fugitive channels	Pneumatic, hydraulic, fluidic	Endoscopic, ultrasound, marker-based	Freeform channels; compliant devices	Bath removal; lumen collapse; contamination	Continuum tools, biopsy/injection channels
Coaxial/core–shell	Hydrogel shells with drug or nanoparticle cores	Swelling, release, magnetic	Optical, MRI/MPI	Compartmentalized payload; tunable barrier	Shell defects; cross-contamination; diffusion	Localized and sequential delivery
Field-assisted/post-magnetized	Hard- or soft-magnetic particle composite	Programmed magnetic torque/force	MRI/MPI where separately validated	Encoded domains; untethered motion	Field depth; stiffness; heating; aggregation	Microrobots, deployable structures
Molding-assisted/hybrid	Printed molds or rigid interfaces with cast soft body	Pneumatic, hydraulic, cable	MRI, endoscopic, X-ray/ultrasound	Clinical scale; integrated hardware	Printing may not define active body; interfaces	Adjacent biopsy and access comparators

**Note:** Route selection should follow the intended clinical function and be validated with the complete material–process–device system.

**Table 5:** Stimulus-responsive mechanisms and physiological validation requirements.

Stimulus	Representative Smart Material	Mechanism	Physiological Relevance	Required Validation
Hydration/ions	Ionic or anisotropic hydrogels	Differential swelling, bending, pore opening	Strongly medium-dependent	Serum/salt/mucus response; kinetics; reversibility
pH	Ionizable hydrogel or linker	Protonation-driven swelling or cleavage	Tumor pH is heterogeneous and gradients may be small	Measured local pH; release under flow; off-target leakage
Enzyme	Gelatin/peptide or degradable crosslinks	Cleavage, erosion, release	Enzyme type and activity vary by tissue	Human-relevant activity; selectivity; degradation products
Temperature	PNAGA, PNIPAM, SMP	Phase transition, swelling, shape recovery	Activation above 37 °C can heat healthy tissue	Transition in biofluid; CEM43; perfused models
Light	Au, PDA, CNT, graphene, MXene	Photothermal heating, recovery, gate opening	Limited depth unless endoscopic/fiber access	Wavelength, irradiance, depth, CEM43, photostability
Magnetic field	SPION or hard-magnetic composite	Torque, gradient force, AMF heating	External hardware and working distance constrain dose	Field/gradient/frequency, workspace, heating, retrieval
Electric field	Conductive/ionic composites, LCE	Joule heating, electroactive deformation	Insulation and wet-current safety required	Current density, leakage, electrochemistry, tissue contact
Ultrasound	Porous or self-folding carriers	Acoustic release, vibration or heating	Attenuation and cavitation vary with tissue	Pressure, frequency, cavitation, thermal and mechanical dose

**Note:** PNIPAM, poly(N-isopropylacrylamide). Trigger feasibility must be demonstrated in route- and tissue-relevant conditions.

**Table 6:** Representative diagnostic systems, scope classification and evidence gaps.

Diagnostic Task	Representative System	Role of Printing/Nanomaterial	Guidance or Readout	Highest-Priority Validation
Breast lesion targeting and biopsy	Stormram 4 MR-safe robotic system [68]	3D-printed nonconductive components; pneumatic positioning	MRI guidance; target and needle placement	Deformable-tissue accuracy; MRI artifact; specimen adequacy; workflow comparison
Peripheral lung access	Soft robot for peripheral lung cancer [69]	Miniature pressure-driven continuum body with camera	Distal steering, stabilization, puncture, injection	Respiratory motion; mucus; biopsy yield; sterilization; channel compatibility
Millimeter-scale biopsy	Soft biopsy robot [70]	Miniaturized print-enabled soft mechanism	Tissue sampling under external visualization	Sample mass; target error; puncture force; in vivo trauma
Motor-free detection/surgery	Portable soft robotic arms [71]	Soft arms and hybrid printed components	Cancer detection, manipulation, injection or printing	Oncology-specific diagnostic accuracy; force limits; comparison with standard tools
Fluorescent localization	Printable fluorescent hydrogel; Cy5 system [72,73]	Optical agent confined in soft matrix or contact surface	Endoscopic or intraoperative visualization	Specificity; signal-to-background ratio; depth; bleaching; marker retention; field registration
Magnetic localization	Iron-oxide/TriMag-type printed bodies [11]	Magnetic nanoparticles integrated into printed robot	MPI localization, with actuation validated separately	Dynamic localization error; fragment detection; in vivo dose and clearance
Tumor stiffness mapping	Proposed printed conductive or ionic distal tip (conceptual)	Multimaterial sensor–actuator architecture	Force, impedance or stiffness map registered to anatomy	Histology-linked sensitivity/specificity; depth; curvature and hydration confounds

**Note:** Systems are included as core evidence or explicitly labeled adjacent comparators; inclusion does not imply clinical validation.

**Table 7:** Representative therapeutic systems and translational requirements.

Therapeutic Mode	Representative System	Payload/Active Phase	Navigation or Trigger	Current Evidence	Priority Requirement
Theranostic cargo delivery	Biodegradable 3D-printed microswimmer [12,13]	Iron oxide, targeting chemistry, therapeutic cargo	Magnetic navigation; enzyme-mediated degradation	Cancer cell/microfluidic evidence	Dose per robot; biodistribution; protein corona; in vivo targeting
Adaptive shape-morphing treatment	Microcrab or pH-responsive body [14]	Doxorubicin or particle cargo in responsive network	Chemical trigger plus local positioning	Localized cancer cell treatment	Tumor retention; trigger heterogeneity; systemic delivery
Drug release plus hyperthermia	Degradable magnetic microrobot [15]	Chemotherapy plus magnetic nanoparticles	Rotating/gradient field; AMF heat	In vitro therapeutic effect	Thermal dose; off-target heating; field depth
Ultrasound-triggered therapy	Porous/self-folded robot [16,21]	Drug in porous or folded compartment	Magnetic navigation; ultrasound release	Rapid controlled release	Acoustic pressure in tissue; premature release; cavitation safety
Image-guided retrievable delivery	Self-rolled robot [17]	Contrast and photothermal/drug cargo	Magnetic/X-ray guidance; NIR; retrieval	Integrated workflow demonstration	Retrieval reliability; vessel injury; residual material
Sequential/combination delivery	Helical and multidrug robots [18,19,22]	Magnetic particles plus two or more drugs	NIR, focused ultrasound or staged release	In vitro/preclinical evidence	Dose accounting; trigger orthogonality; combination controls
Thermosensitive release	PNAGA hydrogel robot [20]	Temperature-responsive hydrogel and anticancer drug	Thermal transition	Cancer cell therapy study	Physiological transition; deep-tissue triggering; thermal safety
Chemoembolization	Adjacent nonprinted multifunctional liver microrobot [79]	Embolic/chemotherapeutic payload with imaging markers	Magnetic guidance; X-ray and MRI	Advanced preclinical integration	Comparative efficacy; non-target embolization; catheter workflow
Integrated imaging plus hyperthermia	TriMag microrobot [11]	Magnetic nanocomposite	Actuation, MPI and AMF treatment	Multifunctional printed platform	In vivo localization; temperature coverage; clearance

**Note:** Evidence levels are study-specific. Diagnostic visibility, actuation, release and treatment should be validated as separate functions before an integrated therapeutic claim is made.

**Table 8:** Comparison of imaging modalities for soft and miniature robotic systems.

Modality	Typical Spatial Resolution	Penetration	Real Time	Robot Visibility	Advantages	Limitations
Fluorescence (including endoscopic)	~1–100 µm locally; system-dependent	mm to cm; surface/endoluminal	Yes	Direct with stable fluorophore/particle	High frame rate; molecular labels	Limited depth; bleaching; background
Ultrasound	~50 µm–1 mm	Several cm	Yes	Geometry/echogenic marker-dependent	Portable; nonionizing; tissue motion	Angle, shadowing, operator dependence
Photoacoustic	~50 µm–1 mm	mm to several cm	Often near-real-time	Absorber-dependent	Optical contrast at greater depth	Fluence/attenuation; limited clinical integration
X-ray/fluoroscopy	~0.1–1 mm	Whole-body projection	Yes	Requires radiopaque marker	Established interventional workflow	Ionizing radiation; weak soft-material contrast
CT	~0.3–1 mm	Whole body	Intermittent	Requires radiopacity/contrast	3D anatomy; quantitative attenuation	Radiation; limited continuous feedback
MRI	~0.5–2 mm; sequence-dependent	Deep/whole-organ	Usually limited frame rate	SPION marker or indirect anatomy	Soft-tissue contrast	Artifacts; bore/access; MR safety classification
MPI	~0.5–5 mm; system-dependent	Deep; no tissue background	Potentially real-time	Direct SPION tracer signal	Potentially quantitative	Limited clinical availability; field integration

**Note:** Ranges are indicative and depend on the system, target depth, acquisition parameters, reconstruction and marker design [38]. Compatibility must be demonstrated for the complete robot–scanner–actuation configuration.

**Table 9:** Translation questions relative to current standards of care.

Clinical Use Case	Current Standard	Author-Proposed Robotic Value to Be Tested	Required Comparative Endpoints
Breast lesion biopsy	MRI-guided core-needle/vacuum-assisted biopsy	Softer patient-proximal positioning; reduced bore hardware	Target error, artifact, specimen adequacy, time, MR Safe/Conditional labeling
Peripheral lung lesion	Navigational bronchoscopy, robotic bronchoscopy, CT-guided biopsy	Smaller compliant distal access; stabilization; same-tool injection	Reach, pneumothorax/bleeding, diagnostic yield, sample quality, procedure time
Gastrointestinal/surgical localization	Endoscopy, fluorescence guidance, intraoperative ultrasound	Contact-based local sensing or carrier tracking	Specificity, depth, registration, false positives, workflow burden
Focal liver therapy	Transarterial chemoembolization/ablation	Magnetically guided distribution and multimodal tracking	Tumor coverage, non-target embolization, dose, radiation, survival and complications
Local drug delivery	Injection, implant, hydrogel/depot, catheter	Programmable placement, trigger, retrieval	Delivered dose, leakage, pharmacokinetics, therapeutic index, retrieval/degradation
Hyperthermia	Clinical radiofrequency, microwave, laser or magnetic heating	Localized mobile heat source	CEM43 coverage, healthy-tissue dose, hardware power, temperature monitoring

**Note:** The comparator should match the proposed route, lesion type and procedural objective.

**Table 10:** Minimum reporting and validation framework for 3D/4D-printed nano-enabled soft robots in oncology.

Domain	Minimum Measurements	Reproducibility/Statistics	Controls/Comparator
Formulation	Composition; nanoparticle size/surface/loading; rheology/cure; drug state	Author-proposed baseline: at least 3 independent batches; justify alternatives; prespecify acceptance limits	Blank formulation; free drug/nanoparticle
Manufacture	Process window; yield; dimensions; defects; interfaces; sterilization	Batch and within-batch variance; operator/instrument recorded	Unprinted/cast comparator where relevant
Actuation	Field/input; workspace; force/torque/speed; payload; confinement	Independent robots and batches; uncertainty and failure rate	No-field; unloaded robot; conventional tool
Imaging	Resolution; depth; frame rate; artifact; localization error/latency	Blinded analysis where possible; calibration drift	Marker-free; static target; anatomy-only
Drug delivery	Loading; encapsulation efficiency; leakage; release under flow; delivered/residual dose	Dose normalization; kinetic model and confidence intervals	Free drug; carrier; unloaded/trigger-only controls
Heating/ultrasound	3D temperature; CEM43; field/irradiance/acoustic dose	Sensor uncertainty; spatial/temporal repeatability	Energy only; particles only; sham exposure
Diagnosis	Target error; specimen adequacy; signal-to-noise ratio; sensitivity/specificity; time	Ground truth; blinded scoring; appropriate sample size/statistics	Standard biopsy/imaging workflow
Biological safety	Cytotoxicity; hemocompatibility; histology; immune/thrombotic effects	Exposure-matched acute and longitudinal endpoints	Material/drug/energy controls; healthy animals/tissue
Fate	Retrieval success; fragmentation; degradation; biodistribution; excretion	Mass balance and time course; residual-material detection	Passive carrier and free-nanoparticle controls
Translation	Scale-up; shelf life; durability; power; thermal management; human factors	Risk analysis; lot release; comparator workflow and benefit	Current standard of care

Note: This framework is an author-proposed minimum reporting set, not a universal regulatory checklist. Numeric suggestions, including the baseline of at least three independent batches, are engineering recommendations intended to expose between-batch variation and are not regulatory requirements. Biological evaluation, sterilization and risk controls should be selected for the intended contact, route, duration and risk profile with reference to applicable standards [84,85,86,87].

## Data Availability

No new data were created or analyzed in this study. Data sharing is not applicable to this article.

## Abbreviations

2PP, two-photon polymerization; 3D/4D, three-dimensional/four-dimensional; AMF, alternating magnetic field; CEM43, cumulative equivalent minutes at 43 °C; CNT, carbon nanotube; CT, computed tomography; DIW, direct ink writing; DLP, digital light processing; GelMA, gelatin methacryloyl; LCE, liquid-crystal elastomer; MPI, magnetic particle imaging; MRI, magnetic resonance imaging; NIR, near-infrared; PDA, polydopamine; PEGDA, poly(ethylene glycol) diacrylate; PNAGA, poly(N-acryloyl glycinamide); QD, quantum dot; SLA, stereolithography; SMP, shape-memory polymer; SPION, superparamagnetic iron oxide nanoparticle; UCNP, upconversion nanoparticle.
